# Thermal Sensor Allocation for Effective and Efficient Heat Transfer Measurements in Transportation Systems

**DOI:** 10.3390/s23052803

**Published:** 2023-03-03

**Authors:** Jorge Saavedra, David Gonzalez Cuadrado

**Affiliations:** 1 European Institute For Aviation Training and Accreditation (EIATA), Universidad Rey Juan Carlos, Fuenlabrada, 28943 Madrid, Spain; 2 Gas Turbine Lab, Massachusetts Institute of Technology, 70 Vassar Street, Cambridge, MA 02139, USA

**Keywords:** heat transfer, thermal sensors, cooling, sensor allocation, heat conduction, global optimization, uncertainty evaluation

## Abstract

Power plants, electric generators, high-frequency controllers, battery storage, and control units are essential in current transportation and energy distribution networks. To improve the performance and guarantee the endurance of such systems, it is critical to control their operational temperature within certain regimes. Under standard working conditions, those elements become heat sources either during their entire operational envelope or during given phases of it. Consequently, in order to maintain a reasonable working temperature, active cooling is required. The refrigeration may consist of the activation of internal cooling systems relying on fluid circulation or air suction and circulation pulled from the environment. However, in both scenarios pulling surrounding air or making use of coolant pumps increases the power demand. The augmented power demand has a direct impact on the power plant or electric generator autonomy, while instigating higher power demand and substandard performance from the power electronics and batteries’ compounds. In this manuscript, we present a methodology to efficiently estimate the heat flux load generated by internal heat sources. By accurately and inexpensively computing the heat flux, it is possible to identify the coolant requirements to optimize the use of the available resources. Based on local thermal measurements fed into a Kriging interpolator, we can accurately compute the heat flux minimizing the number of sensors required. Considering the need for effective thermal load description toward efficient cooling scheduling. This manuscript presents a procedure based on temperature distribution reconstruction via a Kriging interpolator to monitor the surface temperature using a minimal number of sensors. The sensors are allocated by means of a global optimization that minimizes the reconstruction error. The surface temperature distribution is then fed into a heat conduction solver that processes the heat flux of the proposed casing, providing an affordable and efficient way of controlling the thermal load. Conjugate URANS simulations are used to simulate the performance of an aluminum casing and demonstrate the effectiveness of the proposed method.

## 1. Introduction

The operational envelope and autonomy of transportation systems are restrained by the energy sources present in the vehicle. The power plant generally presents the greatest energy and power requirement. Similarly, the power electronics to control, guide, and monitor the overall health of the vehicle may become relevant power demand units. With the current internet of things approach and digital twin implementation, the transportation systems are clustered with power electronics and battery storage and management units [1,2]. As the mission demands progress, the onboard electronics, energy storage systems, and power plants are further burdened, enabling the formation of local hot spots that may endanger the vehicle endurance and its performance. Furthermore, the inclusion of high-power-density electric motors for actuators or propulsion needs increases the internal thermal load in the airframe [3]. Consequently, to prevent the harmful effects of improper thermal management, adequate cooling systems are designed [4,5]. However, the usage of cooling systems generates a supplementary energy demand. Tanda presented various cooling approaches that could be easily integrated into the ground and aerial transport vehicles [6]. Based on the actual thermal response to the present heat sources and the power demand of the different systems on top of the cooling requirements, the operability of the transportation system is constrained.

On the other hand, before introducing refrigeration into the system, one must accurately estimate the thermal load that the given machine is operating under. Otherwise, the amount of coolant could be excessive, draining unnecessarily the energy sources, or insufficient, harming the endurance of the overall system [7]. As stated by Barroso et al. and Zator et al., accurate heat flux loads are essential in designing optimal cooling approaches and their efficient exploitation to guarantee extended operations and minimal maintenance burdens [8,9].

There are multiple heat transfer estimation procedures, such as the one based on the Kutateladze–Leontiev approach described by Plotnikov et al. [7]. Considering natural or forced convection heat influxes, the most extended approach is the one based on dual-sided thermal measurements [8]. Taking thermal evolution measurements on both sides of the material and computing the heat conduction between both boundaries, the heat flux can be accurately estimated. However, this approach requires measuring the temperature on both sides of the material, which could be complex considering the compact internal assembly of power plants, power electronics, or energy storage systems. In this line, the accuracy of the wall temperature measurements is essential to guarantee proper heat transfer computations, as presented by Korobiichuk and A. Ilchenko [5].

Considering the thermal load of power electronics, electric engines, or power plants, the external surface temperature could be easily measured by IR thermography or uniformly spatially discretized local thermometers, such as thermocouples, resistive thermal detectors, or single-sided thin-film sensors. In fact, considering a minimum casing thickness of 5 mm, the outer surface suffers a low-frequency temperature variation. On the other hand, the inner surface, directly exposed to the heat source, may suffer stronger and faster temperature variations. Hence, the inner sensors must be of higher temporal resolution and higher accuracy. Unfortunately, the lower clearance on the inner surface to the surrounding elements hinders the installation of the sensing elements. In this line, in an attempt to mitigate the continuously growing number of sensors [2] and minimize the sensing installation assembly complexity, the sensor allocation must be optimized. Similar approaches are proposed by Mano et al. [10] and Ipaye et al. [2] for air pollution monitoring and mobile crowd sensing, respectively, where optimal sensor allocation is essential to reduce the complexity and cost of the experimental hardware.

During vehicle service, the components are susceptible to mechanical and thermal failures from excessive thermal loads. Furthermore, the instability or high-frequency variation of the heat flux may promote thermal fatigue failure modes that further constrain the operational life of the designs. During such fluctuations, the thermal load is time-dependent; hence, to prevent excessive heat flux levels or thermal fatigue, it is fundamental to provide temperature and heat flux measurements with sufficient accuracy and temporal resolution. However, accurate heat flux measurements may not be enough for achieving such a goal [11]. In addition to the heat flux, the measurement apparatus must consider the identification of the adiabatic wall temperature and, in particular, the adiabatic wall heat transfer coefficient when dealing with convective heat flux to effectively abate or mitigate the forced convection process.

As previously discussed, accurate thermal load estimation is fundamental for efficient cooling scheduling and, hence, minimal energy consumption. However, in general, regardless of the method employed to identify the heat flux, it is essential to dispose of surface temperature measurements. Thus, the hardware must be equipped with complex and expensive instrumentation to monitor the surface temperature, while difficult postprocessing techniques must be applied to compute the heat flux. In line with these requirements, this manuscript presents an approach to derive the thermal load based on the efficient use of thermal sensors, minimizing the hardware requirement while providing a simple and effective way of estimating the heat flux.

This article proposes a methodology to determine the optimal number of sensors required to compute the heat flux over a thin slab, where a heat source is placed over the interior surface, modeling the temperature evolution in the casing of a power electronic or a power plant with local hot spots. Numerical experiments consisting of conjugate unsteady Reynolds average simulations provide thermal surface evolutions to test the sensor distribution and heat flux computation while optimizing the accuracy of the approach. The heat flux estimation procedure is coupled with a global optimizer towards the identification of the optimal number of sensors and their distribution. This procedure allows the use of minimal resources, while guaranteeing accurate heat flux measurements.

## 2. Materials and Methods

Heat flux can be computed based on wall temperature measurements, taking advantage of the impulse response method presented by Oldfield [12] or solving the inner heat conduction as stated by Saavedra et al. [13]. The heat flux is derived from the temperature spatial distribution across the solid thanks to the heat conduction equation:

(1) $$\frac{1}{\alpha }\frac{\partial T}{\partial t}=\frac{\partial^{2}T}{\partial^{2}y}$$

The current methodology assumes constant material properties; however, temperature dependence models can be introduced to reflect the thermal conductivity and heat capacity variation with the temperature of the given substrate. In this case, the modeled casing suffers at most temperature variations of 30 K; hence, the thermal conductivity and heat capacity remain within 1% of their original value. As an attempt to verify the impact of the solid substrate thermal properties’ variation with temperature, the model has been updated including the variation of thermal conductivity and specific heat capacity with temperature [14,15]. One of the datasets was analyzed with the updated model, revealing a heat flux magnitude variation below 0.1%. This variation is mainly promoted by the thermal conductivity variation next to the surface, where the largest temperature variations are observed. However, given the reduced temperature increment, the material properties are minimally altered and, consequently, their impact on the ultimate heat flux estimation is negligible. The consideration of the material properties’ variation will be essential for accurate heat flux estimations if stronger thermal gradients are present in the solid.

To solve the internal heat conduction, the model requires the prescription of the lateral, upper, and lower temperatures. The lateral walls are assumed adiabatic, while the upper and lower surface temperatures are inputs based on the surface monitoring approach. Given the upper and lower boundary conditions with enough temporal resolution, the unsteady heat flux equation can be accurately computed using a Crank–Nicholson numerical discretization [16]. The solution consists of a blend of the forward Euler method at *n* and the backward Euler method *n + 1*:

(2) $$T_{i}^{n+1}=\frac{T_{i}^{n}}{\Delta t}+\frac{1}{2}\left(F_{i}^{n+1}\left(T,\;x,\;t,\frac{\partial T}{\partial t},\frac{\partial T^{2}}{\partial x^{2}}\right)+F_{i}^{n}\left(T,x,\;t,\frac{\partial T}{\partial t},\frac{\partial T^{2}}{\partial x^{2}}\right)\;\right)$$

**Figure 1:**
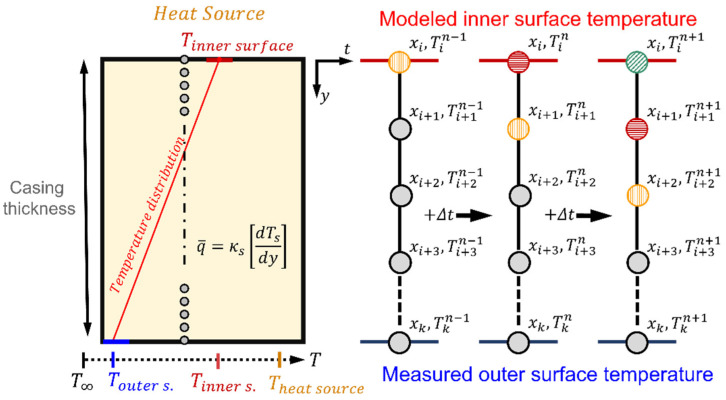
Spatial discretization and numerical scheme representation.

The formulation of this problem is spatially implicit and, given its nonlinear nature, it requires the solution of the complete domain at once. The discretization characteristics of such a scheme are presented in Figure 1.

The complete description of the convective heat transfer required the identification of the heat transfer coefficient, h. In this line, Moffat [17] presented different invariant descriptors that could help describe the convective heat flux. The characterization of the adiabatic wall temperature and the associated adiabatic wall heat transfer coefficient allows the accurate measurement of the heat flux loads under various thermal boundary conditions, which could be extremely valuable in set-ups with unsteady operating conditions. However, to correctly identify both adiabatic wall temperature and adiabatic wall heat transfer coefficient, the surface temperature measurements must be performed with enough temporal discretization. This approach has been demonstrated and extensively exploited in gas turbine heat flux measurements of components exposed to periodic oscillation conditions [18] or unsteady operation [19,20]. The use of these invariants enables the complete description of the transient heat flux data, analyzing both its source and its consequences. Similarly, these invariants have been proven resourceful to characterize the heat flux in the electronic industry in geometries exposed to nonuniform and unsteady thermal boundary conditions [21,22].

**Figure 2:**
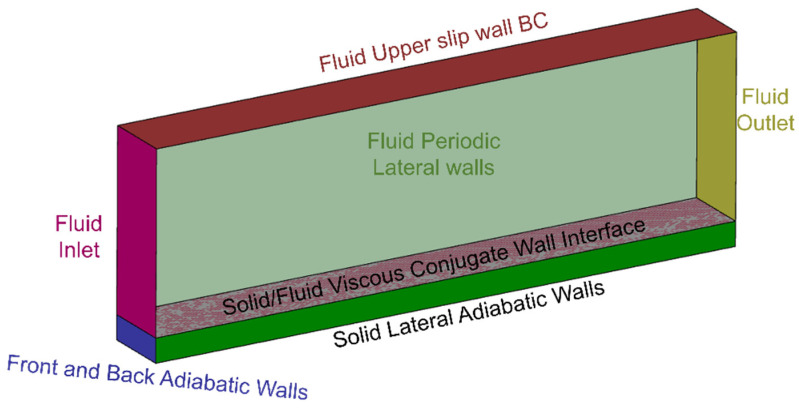
Conjugate unsteady Reynolds average Navier–Stokes numerical domain.

Conjugate unsteady Reynolds average Navier–Stokes simulations are used to generate experimental temperature evolutions to test the computation of heat flux based on discrete thermal sensor allocation. The URANS simulations model the casing considering the inner wall exposed to forced convection conditions and a local hot spot while natural convection is suffered on the opposite side. The domain, presented in Figure 2, is 400 mm long and 30 mm wide. The solid slab has a thickness of 10 mm, while the fluid side of the domain has a height of 50 mm.

The solid was modeled as a slab of aluminum of uniform and constant material properties, while the fluid was modeled as air following the ideal gas equation with the specific heat, thermal conductivity, and molecular viscosity varying with the gas temperature. The inlet assumed a cantilever slab configuration, developing the boundary layer from the domain inlet and flow was vented through the outlet, modeled as a static pressure boundary condition. The inlet imposed a flow entering the domain at 10 m/s and 400 K using a velocity inlet prescription. The fluid lateral walls were modeled as periodic boundaries and the top surface was set as inviscid. The fluid domain height was verified to suffer negligible free-stream variations because of the boundary layer growth over the conjugate surface. The boundary between fluid and solid was modeled as a conjugate viscous wall, while the lateral and frontal sides of the solid were modeled as adiabatic. Ultimately, natural convection was assumed at the lower solid wall, assuming an external temperature of 300 K. The turbulence closure was achieved by means of the k-ω transitional SST model, given its superior performance for confined flows, particularly in the near-wall flow region.

The proposed numerical domain simulates the performance of the external wall of an aluminum enclosure exposed to a local heat source and suffering the passage of a heated stream, such as the ventilation of coolant flow or the power plant secondary exhaust. On the opposite side of the casing, natural convection is assumed to mimic the exposure of the casing to inner airframe stagnant conditions. Following this approach, the casing would be exposed to extreme conditions, suffering considerable thermal load from the stream and local hot spots on one side, while exposed to stagnant conditions on the opposite. The fluid is modeled as air, replicating the operation at low-altitude atmospheric conditions, hence maintaining a Prandtl number around 0.7, indicating the relevance of both thermal and momentum fluid diffusion. In this line, the inner side of the casing exposed to the heated stream suffers the heat signature of the warmed current circulating along its wall. Considering the fluid velocity and gas density over the interior wall, the Richardson number remains below 0.1, describing the dominance of the forced convection in the surface heating process. Consequently, the correct simulation of near-wall flow evolution is fundamental for proper heat load prediction. Since the convective heat flux over the interior surface will be a function of the local adiabatic wall temperature and adiabatic heat transfer coefficient, both parameters will be driven by the total flow temperature and wall-normal velocity and temperature distribution.

On the opposite side, assuming that the casing is exposed to inner airframe stagnant conditions, the Richardson number would be beyond 10, reflecting the minimal influence of forced convection and the prime relevance of the natural convection process. Hence, to reduce the computational effort, a natural convection boundary is directly implemented in the exterior surface of the casing.

**Figure 3:**
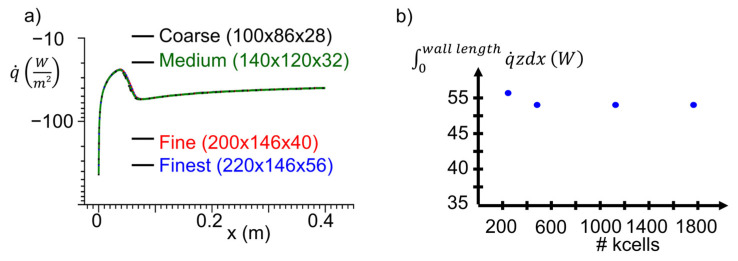
Mesh sensitivity analysis: representation of (**a**) heat flux distribution along the surface and (**b**) heat flux integral over the surface for various spatial resolutions.

The domain was meshed following a structured blocking strategy. The procedure presented by Celik et al. [23] was used to assess the independence of the results from the chosen geometrical discretization. The y+ was kept below 1 for all the fluid vertical surfaces. In this line, Figure 3a,b represent the heat flux distribution along the centerline and the heat flux integral over the conjugate surface for different 3D numerical domain resolutions. Four different grids are compared, presenting only minor differences in the boundary layer transition region. Based on the integral heat load, just the coarse mesh is particularly away from the trend. Following Celik’s procedure, the Grid Convergence Index of the fine grid is below $1\times 10^{-3}$, assuring spatial discretization. The URANS computational approach was verified for a similar geometry, including an orifice for film cooling in a previously presented research publication [24].

A modified numerical domain with a slab thickness of 50 mm was used to verify the current modeling approach. The local wall temperature evolution on the central point of the inner surface was monitored throughout a transient simulation that started with the solid at 300 K. The convective heat transfer coefficient at that point was derived from the CFD heat flux signature and the wall to free stream temperature difference.

The inner and outer surface temperature conditions predicted by the conjugate solver were directly provided to the heat conduction solver. The heat conduction solver and the thermal processing approach were verified against the analytical solution for constant surface convection as described by Bergman et al. [25].

(3) $$\frac{T\left(y,t\right)-T_{i}}{T_{\infty }-T_{i}}=erfc\left(\frac{y}{2\sqrt{\alpha t}}\right)-\left(\exp\left(\frac{hy}{k}+\frac{h^{2}\alpha t}{\kappa^{2}\;}\right)\right)\left(erfc\left(\frac{y}{2\sqrt{\alpha t}}+\frac{h\sqrt{\alpha t}}{\kappa \;}\right)\;\right)$$

**Figure 4:**
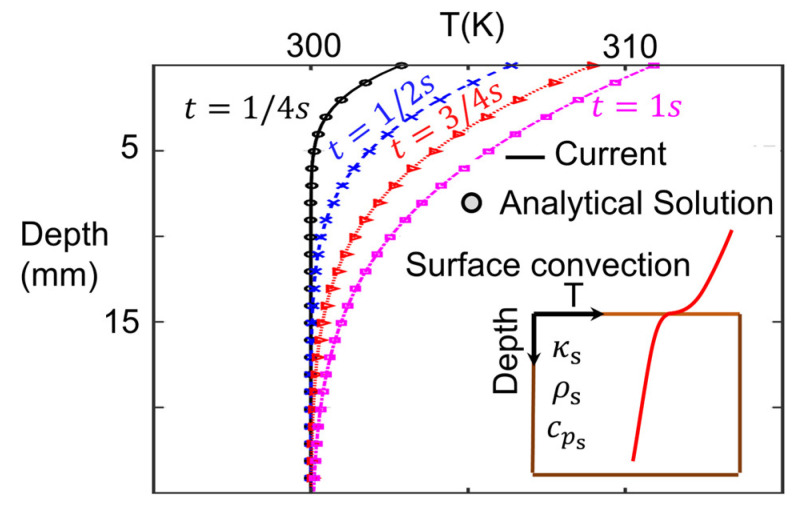
Heat solver verification against analytical surface convection. Solid temperature distribution at different time instances while suffering constant forced convection on the upper boundary, where the symbols are the evaluation of the analytic solution by Bergman et al. [25] and the solid lines are the results of the current methodology.

The derived heat transfer coefficient based on the conjugate simulation, free-stream flow temperature, and initial wall temperature in the central point were prescribed in the analytical solution. Considering the heat transfer coefficient, solid properties, and inner thermal conduction, the analytical solution describes the inner solid temperature distribution along the slab centerline. The analytical solution is compared against the heat conduction solver results for different time instances after the initialization of the simulation in Figure 4. The excellent agreement between both methodologies for all the periods being presented verifies the accuracy of the heat conduction solver.

**Figure 5:**
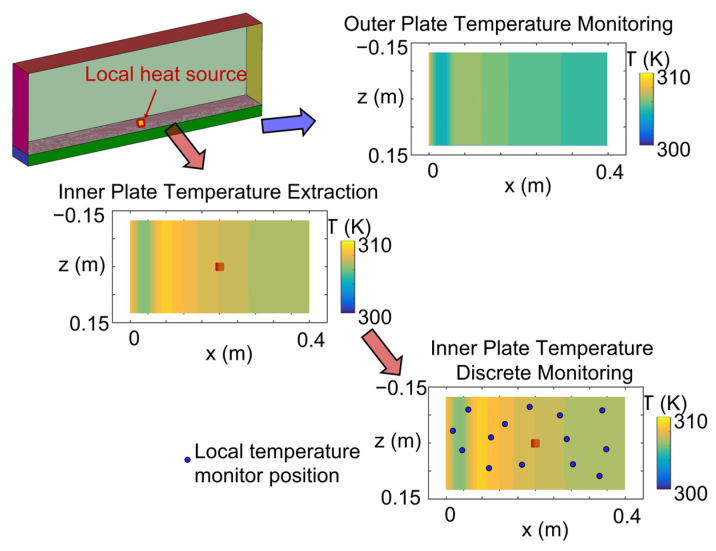
Inner and outer plate surface temperature extraction from conjugate unsteady Reynolds average Navier–Stokes simulations.

To model the impact of a heat source over the inner surface, a local heat source was set in the center of the numerical domain, replicating a hot spot in the interior power electronics. The heat source is directly applied over a confined region by means of a user-defined function that sets a heat source magnitude of 10 kW/m^2^ over 50 mm^2^. The outer wall temperature was directly extracted from the numerical simulations and fed into the heat conduction solver, assuming an easier thermal characterization of this surface. On the other hand, the inner surface temperature was locally sampled at given locations to replicate the measurements of discrete thermal sensors, as depicted in Figure 5.

Before solving the heat conduction across the solid, the temperature distribution over the inner slab surface must be reconstructed. There is a myriad of methods available to interpolate a surface distribution based on discrete measurements, such as polynomial interpolation, radial basis functions, inverse distance weighted, density weighted, artificial intelligence, or Kriging interpolation, as illustrated in [26]. In this procedure, the Kriging interpolation was selected given its easier integration with optimization routines. The Kriging interpolation is based on a statistical approach relying on weighted variance minimizing the error between the actual and the estimated values [27].

The Kriging interpolation is a statistical spatial interpolation method that incorporates information on the spatial variance and covariance of the sampled measured data to predict the research variable in unsampled data points. It uses a limited set of sampled data points to estimate the value of a variable over a continuous spatial field. The Kriging predictor is an “optimal linear predictor” and an exact interpolator, meaning that each interpolated value is calculated to minimize the prediction error for that point. It is a flexible method that can provide anisotropic information of the variable based on weights on standard statistical errors related to spatial measured data. In this case, interested in reconstructing the temperature distribution over the surface *T*, the variable is the surface temperature and the Kriging estimator is expressed linearly as T* [28]:

(4) $$T^{*}\left(x_{o},z_{0}\right)=\sum_{i=1}^{n}\lambda_{i}T\left(x_{i},z_{i}\right),$$

where *T*(*x_i_,z_i_*) are the measured locations and the *λ_i_* are the weights applied to each measurement. These weights are computed based on the statistical properties of the data [29]. To guarantee the convergence, the method computes the residual based on a model m(x,y) that can be based on the main population level 𝜇:

$$Y^{*}\left(x_{i},z_{i}\right)=T\left(x_{i},z_{i}\right)-\mu \left(x_{i},z_{i}\right)$$

and:

(5) $$Y^{*}\left(x_{o},z_{0}\right)=\sum_{i=1}^{n}\lambda_{i}Y\left(x_{i},z_{i}\right),$$

Then, coefficients *λ_i_* are computed by minimizing the expected error variance of the estimate Y^*:

(6) $$e^{2}=E\left[{\left(Y^{*}\left(x_{0},z_{0}\right)\right)}^{2}\right]-2E\left[\left(Y^{*}\left(x_{0},z_{0}\right)\right)\left(Y\left(x_{0},z_{0}\right)\right)\right]+E\left[{\left(Y\left(x_{0},z_{0}\right)\right)}^{2}\right]$$

where the operator E computes the error. Then, introducing the evaluation of Y*:

(7) $$e^{2}=\sum_{i=1}^{n}\sum_{j=1}^{n}\lambda_{i}\lambda_{j}E\left[Y\left(x_{i},z_{i}\right)Y\left(x_{j},z_{j}\right)\right]+2\sum_{i=1}^{n}\lambda_{i}E\left[Y\left(x_{i},z_{i}\right)Y\left(x_{j},z_{j}\right)\right]+C\left(0\right)$$

Being the covariance defined as a function of x increments (δh):

(8) $$C\left(\delta h\right)=\frac{1}{m}\sum_{i=1}^{m}\left[Y\left(x_{i}+\delta h\right)Y\left(x_{i}\right)\right]$$

Taking advantage of the local sensors, the covariance can be computed, leading to the identification of the coefficients *λ_i_*. After minimizing the variance error:

(9) $$\frac{\partial e^{2}}{\partial \lambda_{i}}=2\sum_{j=1}^{n}\lambda_{j}C\left(x_{i}-x_{j}\right)=C\left(x_{i}-x_{0}\right)\;$$

The procedure leads to:

(10) $$\sum_{j=1}^{n}\lambda_{j}C\left(x_{i}-x_{j}\right)=C\left(x_{i}-x_{0}\right)\;$$

Finally, to close the equations, an additional constraint must be imposed. In this case, the sum of all the coefficients of the weights is set to be unitary. Therefore, the system of equations to calculate the weights results in:

(11) $$\sum_{j=1}^{n}\lambda_{j}C\left(x_{i}-x_{j}\right)=C\left(x_{i}-x_{0}\right)\;for\;i=1,2,3,\ldots .n\;\sum_{j=1}^{n}\lambda_{j}=1$$

**Figure 6:**
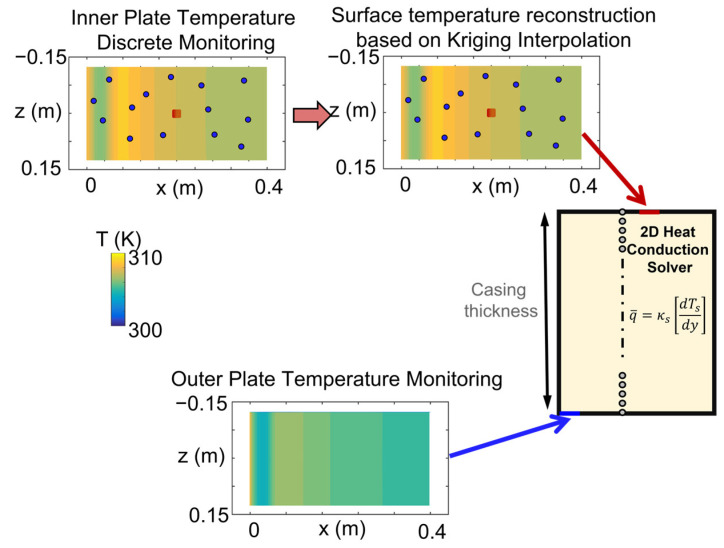
The locally monitored upper temperature evolution is introduced in the Kriging interpolator to reconstruct the surface temperature distribution and then fed along with the lower temperature distribution to the conduction solver for heat flux estimation.

The result of this interpolation is the value of the temperature distribution at the nonmeasured areas and an indicator of the error variance. Considering the procedure, the precision of the Kriging interpolation reconstruction is strongly influenced by the sensor allocation. Figure 6 depicts the data prescription for the heat conduction solver.

## 3. Results

**Figure 7:**
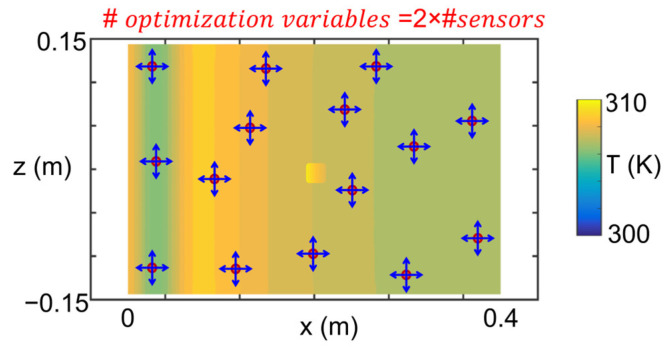
Sensor allocation design variables representation.

For each sensor distribution, the Kriging surface temperature reconstruction is compared against the real surface temperature extracted from the numerical experiments. The heat flux computation is strongly dependent on the accurate thermal measurement; hence, the thermal load accurate computation requires appropriate inner surface temperature reconstruction. There are two design variables for each sensor placed on the inner surface, as illustrated in Figure 7.

**Figure 8:**
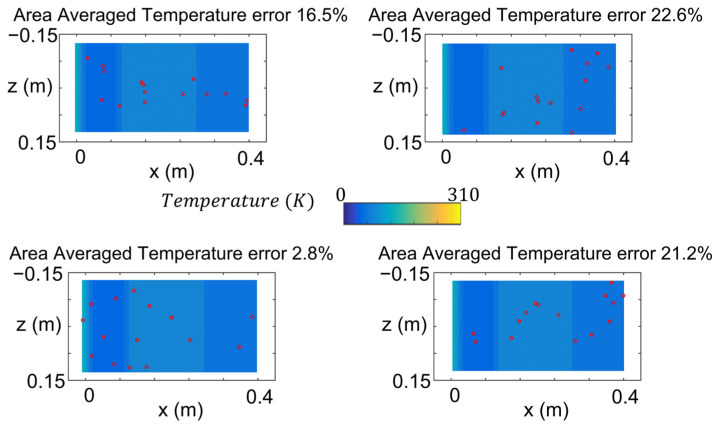
Area-averaged temperature reconstruction error for different sensor allocation distributions.

To identify the optimal location of the thermometers, the sensor coordinates are defined as the design variables of an optimization procedure that pretends to minimize the difference between the Kriging reconstructed temperature and the actual surface temperature distribution. The optimizer uses a simulated annealing procedure to identify the best co-ordinates to place each sensor [30,31]. Figure 8 summarizes the area average temperature error of the reconstruction for four different sensor spatial distributions, considering the use of 16 sensors, hence 32 design variables.

(12) $$area\;average\;temperature\;error=\frac{\iint_{S}^{}T_{kriging}dxdz-\iint_{S}^{}T_{URANS}dxdz}{\iint_{S}^{}T_{URANS}dxdz}$$

The temperature reconstruction accuracy, as previously stated, is extremely sensitive to the allocation of the thermal sensors that are fed into the interpolator. Using the same number of sensors, the error on the reconstruction can range from 40% down to 1%. The cases presented in Figure 8 represent cases of poor accuracy with errors above 15%. Four different cases are summarized in Figure 8. The different subfigures represent the same surface temperature with different sensor distributions.

The area-averaged error, computed as the difference between the Kriging reconstruction using those sensors and the actual wall temperature, is summarized in each subfigure’s title. When the local sensors are clustered in regions with uniform temperature distribution, for instance, the one displayed in the upper right corner, the reconstruction is inaccurate. Similarly, if the sensors are centered in the domain without enough spatial resolution in the regions with strong gradients the accuracy remains insufficient, as in the upper left case. In this line, even if uniformly distributed over the test article surface, without proper resolution in the strong gradient regions x ≈0.01 m & x ≈0.1 m, the error remains beyond 20%, as illustrated in the lower right case. Those cases tend to have the sensors clustered areas of the domain, hence being unable to accurately model the overall distribution of temperature. On the other hand, the case illustrated displayed in the lower left corner has the sensors distributed along the complete width and length of the surface and particularly clustered in the regions with stronger spatial gradients. Relevant distribution characteristics were fed to the Kriging interpolator. Therefore, the interpolator achieves a much better reconstruction, leading to an error below 3%.

**Figure 9:**
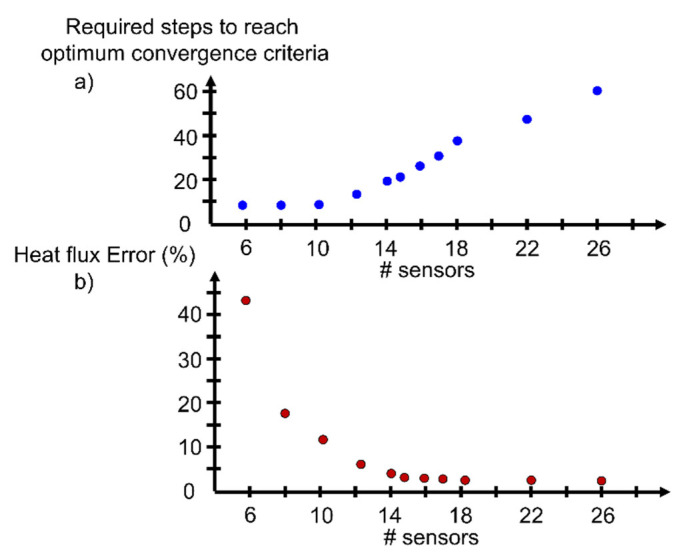
(**a**) Computational cost: optimization steps required to achieve global optimizer convergence; (**b**) heat transfer estimation error for various number of sensors placed in the optimal location to achieve maximum possible precision.

The optimizer leads to the location where the discrete temperature readings lead to the most accurate surface reconstruction and, hence, the most precise heat flux estimation. Taking advantage of the Kriging interpolator coupled with the simulated annealing optimizer, the thermal sensor allocation required to accurately measure the heat flux can be computed. Considering both the number of sensors and their given spatial distribution, Figure 9b represents the error achieved on the heat flux computation for the different number of sensors distributed along the inner casing surface. In each case, the optimal sensor location to lead toward an accurate heat flux estimation must be identified by the optimizer. As the number of sensors increases, the computational burden to identify the correct sensor placement grows quasi-exponentially. Figure 9a illustrates the number of simulated annealing optimization steps required to reach the optimal convergence criteria.

For a reduced number of sensors, the optimizer identifies the optimal spatial and spanwise sensor positions in less than 20 steps. However, as the number of sensors increases, the optimizer requires up to 60 steps for 26 sensors, implying 52 design variables. For this case, considering a slab of 0.5 m × 0.3 m × 0.01 m, the optimal sensor allocation result is reached once 14 or more sensors are introduced. Beyond the use of 14 sensors, the heat flux computation error suffers minor improvements, while the computational burden and measurement system cost rise. On the other hand, when introducing less than 12 sensors, the error on the heat flux computation rapidly bursts, indicating that there is a minimal number of sensors required to accurately compute the thermal load of the casing. The eventual reconstruction veracity will be driven by the thermal measurement accuracy. The preciseness and uncertainty of the thermal sensor used to monitor the discrete surface temperature evolution will limit the accuracy of the final surface reconstruction using the Kriging interpolator. However, for a given number of sensors, with their corresponding accuracy, this procedure identifies the optimal location to place the sensors toward the most correct reconstruction possible with the available hardware.

**Figure 10:**
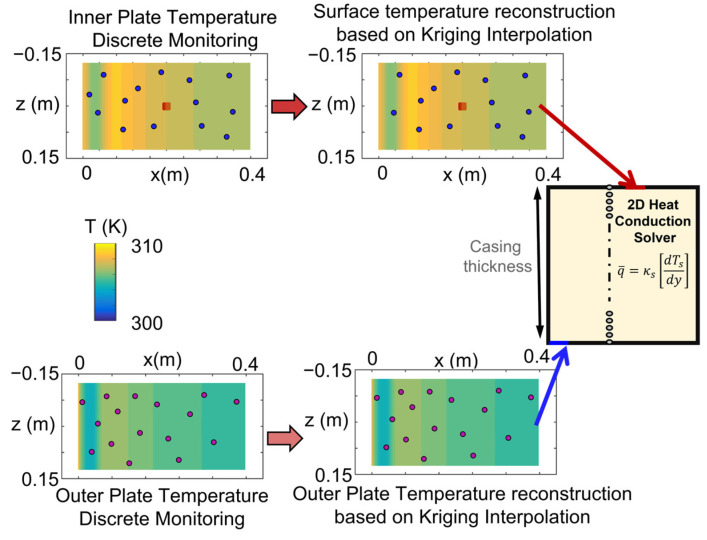
Updated boundary conditions: both surface temperature evolutions are locally monitored and introduced in the Kriging interpolator to reconstruct the surface temperatures and then fed to the conduction solver for heat flux estimation.

Additionally, as identified in the trend displayed in Figure 9b, the number of sensors required to achieve null heat flux error will be infinity, assuming exact temperature measurements and heat flux derivation. Only once the surfaces are fully resolved via local monitors, the reconstruction will be a perfect reflection of the surface evolution without the need for the interpolator. In this line, the proposed methodology helps define the location to install a discrete number of sensors while maintaining reasonable surface thermal load descriptions. Given the number of sensors available, the optimizer can identify the optimal locations to install them on the inner surface to provide a proper surface reconstruction. In the original approach, the outer wall was continuously monitored, assuming the use of infrared imaging in the experimental apparatus. However, given the accurate results provided by the Kriging surface temperature reconstruction, the complete outer surface temperature monitoring is replaced by discrete thermal sensors. The temperature readings of the outer sensors are then fed to the Kriging interpolator and the outer surface temperature distribution is reconstructed. This updated approach replaces the complete outer surface temperature monitoring by distributed local wall temperature measurements, considerably reducing the cost of the experimental hardware and simplifying the postprocessing sequence. Figure 10 summarizes the updated thermal boundary conditions that are now given to the heat flux calculator.

However, as previously stated, the estimated heat flux accuracy depends on the wall temperature boundaries, hence, on the surface temperature reconstruction precision. Thus, the outer surface local thermometers must be strategically placed to deliver a proper temperature reconstruction. To achieve that objective, the optimizer was modified to identify the streamwise and spanwise location of the sensors over the inner and outer surface, while minimizing the surface average temperature reconstruction error. The optimizer looks for the sensor distribution that minimizes the overall error, hence, the addition of the error on each surface. The optimization routine is given the surface temperature on each extreme of the slab on three different operating conditions and the number of sensors available for each surface. A symmetric approach is proposed, where the same number of sensors are given for inner and outer surfaces.

**Figure 11:**
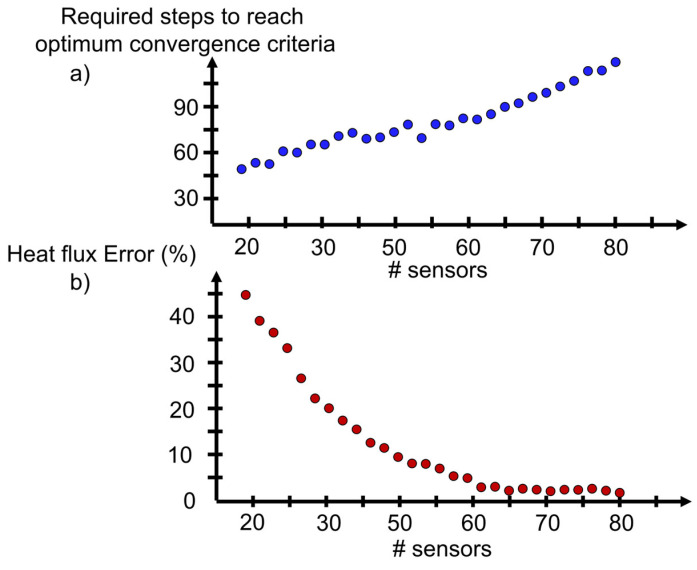
(**a**) Computational cost: optimization steps required to achieve global optimizer convergence, (**b**) heat transfer estimation error for various number of sensors placed in the optimal location to achieve maximum possible precision. Considering discrete sensing and surface temperature reconstruction on both surfaces.

As the number of sensors required to monitor the local temperature on both boundaries increases, the computational burden to identify their optimal location toward accurate surface temperature reconstruction raises, as depicted in Figure 11a. When using beyond 30 sensors the optimizer requires more than 60 steps to identify the streamwise and spanwise location for each thermal reading. Through the different tests performed, the optimizer seems to need about 0.6–1 steps per optimization variable.

In terms of heat flux, Figure 11b represents the error in the heat flux calculation when using the surface temperature reconstruction with an equal number of sensors on each casing boundary. When using less than 20 sensors on each surface, the area average heat flux error based on the heat conduction solver is beyond 10%. As more sensors are dedicated on each surface, the error gradually decays. To achieve heat flux derivation miscalculations below 5%, at least 30 sensors must be placed on each boundary. Ultimately, by allocating 80 sensors, 40 per boundary, the heat flux calculation accuracy is past 99%.

However, given the different temperature distributions on each surface, the use of the same number of sensors on each one of them might not be the most efficient resource assignment. A most efficient thermal sensor allocation might be achieved with an uneven number of sensors on each boundary. In this line, the optimization approach is modified, assigning more variables to the formulation, including the actual number of sensors available for each surface. Therefore, the optimizer must now set the amount of sensing elements on inner and outer boundaries while identifying the streamwise and spanwise location for each one of them towards an accurate surface temperature reconstruction.

**Figure 12:**
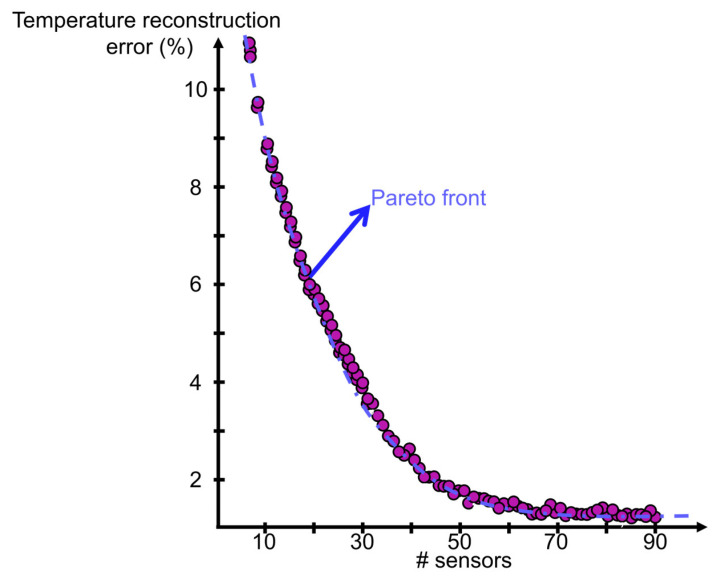
Dual-objective optimization focused on minimizing the number of sensors while optimizing their location towards reducing the temperature reconstruction area average error.

If the number of sensors is not constrained, the optimizer will tend to use as many sensors as possible. Hence, in an attempt to optimize both the accuracy and efficient use of resources, the optimization objective is modified. The renewed target is defined based on the minimization of the temperature reconstruction error while using the minimal number of sensors. The actual optimizer goal is defined as a balance between both minimum objectives. By adjusting the relevance of each one of them the complete optimal space is identified. The results of the dual objective optimization are summarized in Figure 12.

When giving more relevance to the number of sensors restriction, the optimizer finds the location to place each one of them while attempting to achieve the minimal temperature error reconstruction. The variables identifying the number of sensors are constrained as positive integers. For each total number of sensors represented in Figure 12, the numerical procedure identifies different distributions, with an uneven number of discrete measurements on each side. The most accurate temperature reconstruction is always achieved when more sensors are placed on the inner surface. When performing the average across the optimal design space for the different total number of sensors attempted by the optimizer, the best resource allocation requires 65% of the sensors on the inner surface, while only 35% of them are dedicated to monitoring the outer boundary.

The calculated cases are all clustered along the Pareto front. For each combination of sensors attempted, the optimizer finds, taking the required steps, the optimal position to place each local monitor towards a minimal difference between the reconstructed and actual surface temperature. If the global objective is focused on further reducing the reconstruction error, the optimizer allocates a greater number of sensors. In this line, as represented in the Pareto front, to achieve a reconstruction error below 2%, more than 50 sensors must be used. Once more than 70 sensors have been allocated, the accuracy of the temperature reconstruction is beyond 99.5%.

As recognized by the optimization routine, the best sensor allocation is based on denser monitoring over the inner surface. The outer surface requires fewer sensors because the heat conduction across the slab dilutes the spatial temperature gradients that are taking place in the opposite boundary. Hence, the surface temperature distribution on the outer wall has smoother temperature distributions that are easier to reconstruct by the Kriging interpolator.

**Figure 13:**
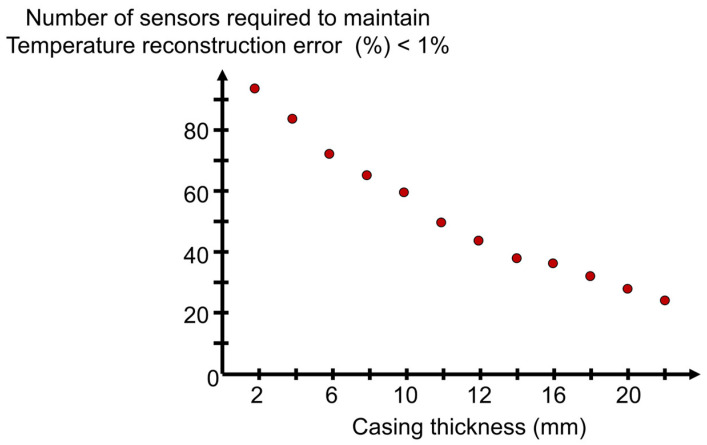
Identification of the optimal number of sensors and their location for various casing thicknesses given a reconstruction error threshold.

Such performance depends on the casing’s thermal conductivity and wall thickness. A casing with higher thermal conductivity will display outer surface temperature patterns like the inner ones, with stronger spatial gradients, hence requiring a more significant number of sensors in the outer boundary. Similarly, the thinner wall thickness will promote similar temperature distributions on both borders. In this line, Figure 13 represents the number of sensors that must be allocated to maintain the temperature reconstruction error below 1% for different casing thicknesses. As the wall thickness decays, more sensors are required to reconstruct the inner and outer surface temperature distributions. Interestingly, as the thickness is reduced, the distribution of sensors between the inner and outer surfaces becomes more symmetric. In fact, the case analyzed with a casing thickness of 2 mm required 94 sensors, 48 being allocated on the inner surface and 46 on the outer one.

As the previous results have demonstrated, having accuracy on the wall temperature boundaries becomes essential to deliver precise thermal load measurements. The accurate assessment of heat flux uncertainty is fundamental to the correct operation of heat exchangers and cooling systems that commonly operate at transient thermal loads that deviate from their design point [32].

The uncertainty can be estimated based on the law of uncertainty propagation considering the influence of the different terms involved in the targeted quantity estimation. This method has been successfully proven in nonlinear mixed-integer mathematical models [33]. In addition, this approach can be tackled either linearly or in combination with a Monte Carlo simulation framework that allows the estimation of higher-order statistical content.

To quantify the uncertainty of the heat transfer measurements, and particularly the influence of the thermal boundary conditions’ reconstruction accuracy, several factors must be considered. The principal sources of uncertainty in the methodology are the wall temperature acquisition, the surface temperature reconstruction, casing thickness, casing material properties, and the heat flux computation method. The following tables summarize the linear uncertainty propagation as described by Moffat [34]. This procedure has been previously used to estimate the propagation of uncertainty in mathematical heat transfer calculations [35,36,37].

Table 1 summarizes the results of the uncertainty propagation estimation. The physical sources of uncertainty are the casing thickness, casing material properties, and wall temperature. The uncertainty analysis is performed based on an aluminum casing with a thickness of 10 mm. Both dimensions’ and material properties’ uncertainties are derived from casing manufacturer reports. The last physical measurement involved in the heat flux calculation is the wall temperature measurement. Assuming that the wall temperature is monitored with thermocouples, an uncertainty of 1.5 K based on linear sensor calibration is proposed. All error evaluations are given at a 95% confidence level.

The wall temperature measurements are fed into the Kriging interpolator to reconstruct the inner and outer surface temperature profiles. The reconstructed profiles are then fed as boundary conditions to the heat conduction solver and, ultimately, the surface heat flux is computed.

The integral heat flux in mean value conditions over both surfaces is 21.65 W, summarized in the lower left corner of the table. Each row in the table presents prime variables for the heat flux calculation. The nominal value and its respective units are summarized in the first two columns of the table. Then, the local uncertainty of that physical quantity, based either on the manufacturer data set or calibrated uncertainty, is defined in the third column. The fourth column indicates the integral heat flux value obtained when altering just the respective prime variable of influence. For instance, by modifying the casing thickness, adding to its nominal value its uncertainty ($1\times 10^{-5}$ m), and considering the other prime variables unaltered, the heat flux in the boundary is recomputed, obtaining an integral value of 21.653 W. The following column represents the percentage of heat flux variation because of the casing thickness uncertainty. Finally, the last column summarizes the sensitivity of the integral heat flux to the variations on each prime variable. The casing properties are homogeneously altered in the numerical domain and the heat flux is derived. Based on the linear uncertainty propagation, the heat flux variation under each individual casing modification is below 0.21%, the thermal conductivity being the most relevant factor.

Ultimately, the local wall temperature measurements fed to the Kriging are randomly altered, with a mean level variation equivalent to the sensing uncertainty. The renewed thermal boundary conditions are introduced in the Kriging interpolator. The reconstructed surface thermal distributions are prescribed as boundary conditions for the heat transfer solver and the heat flux variation is computed at 0.48%, as summarized in Table 1. Combining the uncertainty of the different physical factors, the overall uncertainty of the heat flux calculation is 0.55%, displayed in the lower right corner of the table.

The uncertainty propagation study reveals the sensitivity of the heat flux to the various physical parameters involved, as summarized in the last column of Table 1. In this line, the heat flux calculation is extremely sensitive to the temperature readings. The introduced uncertainty level in that physical parameter is now given based on the sensor accuracy and calibration. However, the heat flux calculator reads the overall surface temperature distribution provided by the Kriging interpolator. Hence, any interpolation or reconstruction error will raise the thermal monitoring uncertainty in the model and boost the heat flux calculation uncertainty. Therefore, accurate thermal descriptions are essential to precise thermal load characterization. In this line, this manuscript presents a cost-effective methodology to identify the optimal thermal sensor allocation toward efficient and accurate heat transfer measurements.

The proposed methodology has been successfully implemented in multiple experimental campaigns in the field of micro-electronics [26,38,39], gas turbines or gas turbine components [36,37,40], and heat exchangers [24]. In the experiments leading toward those publications, the temperature reconstruction based on the Kriging interpolation of discrete sensors placed via optimal position identification was used to derive the heat load. Hence, the application of the presented procedure reduced the number of sensors required for the experiments, which minimized the experimental cost of the campaigns.

## 4. Conclusions

This paper introduces a methodology to allocate the optimal number of thermal sensors required to accurately measure the thermal load of a casing exposed to forced convection and a local hot spot on the inner surface while suffering natural convection conditions on the opposite wall. Proper heat transfer measurements are the fulcrum towards effective and efficient thermal load management. The proposed procedure couples the use of Kriging interpolation, to reconstruct the surface distribution based on discrete measurements, with a simulated annealing optimizer that leads towards the minimal number of sensors required to accurately estimate the heat flux while identifying the co-ordinates where those sensors should be installed.

The heat flux is computed based on the solid heat conduction using the inner and outer casing wall temperature evolution. The outer surface is traditionally easier to thermally characterize and, considering its mild temporal variations, it is commonly easy to identify its overall temperature evolution. On the other hand, the inner casing surface suffers stronger temperature variations and, given the assembly constraints, it is frequently more complex to empirically describe. A dual-objective optimization enables the identification of the minimal number of sensors required to provide an accurate surface temperature reconstruction. Additionally, the optimizer defines the location where each sensor must be placed to provide valuable information to the Kriging interpolation model. The procedure is tested for multiple surface temperature profiles and different casing thicknesses, demonstrating its ability to recognize the different discrete instrumentation needs as the thermal driving effects and mechanisms are altered. This methodology demonstrated that the uncertainty in the heat flux estimation can be reduced by more than 30% for the same number of sensors just by optimizing their allocation.

The proposed procedure provides an efficient and effective approach to minimize the number of sensors required, minimizing the complexity and cost of the sensing apparatus assembly, while guaranteeing accurate heat transfer assessments. The proper thermal reconstruction toward accurate heat flux measurements is demonstrated through an uncertainty propagation analysis.

## Figures and Tables

**Table 1:** Heat flux uncertainty.

	Value	Unit	Uncertainty	$\iint_{S}^{}\dot{q}dS\;(W)$	$\Delta \iint_{S}^{}\dot{q}dS\;\%$	Sensitivity
Aluminum thickness	0.01	m	$1\times 10^{-5}$	21.653	0.01	0.03
Aluminum κ	180	W/m/K	7.2	21.696	0.21	0.05
Aluminum ρ	2700	kg/m^3^	108	21.686	0.17	0.04
Aluminum c_p_	897	J/kg/K	10	21.660	0.05	0.04
Tw	300	K	1.5	21.753	0.48	1.08
$\iint_{S}^{}\dot{q}dS$	21.65	W	Global $\iint_{S}^{}\dot{q}dS$ **uncertainty** 0.55%			

## Data Availability

The data presented in this study are available on request from the corresponding author. The data are not publicly available due to test article geometrical characteristics disclosures limitations.

## Nomenclature

α	Thermal diffusivity (m^2^/s)
$c_{p}$	Specific Heat Capacity (J/(kgK))
δh	Interpolator spatial increments
E	Interpolator error operator
κ	Thermal conductivity (W/(mK))
𝜇	Main population level
ρ	Density (kg/m^3^)
T	Temperature (K)
x,y,z	Spatial coordinates (m)
C	Covariance
Δ	Increment
e	Interpolator expected error variance
h	Heat transfer coefficient (W/(m^2^K))
λ	Interpolator weights
$\dot{q}$	Heat Flux (W/m^2^)
t	Time (s)
T_w_	Wall Temperature (K)
Y	Interpolator residual
